# Temperature-Controlled Synthesis of High-Voltage Spinel LiNi_0.5_Mn_1.5_O_4_ Films via Metal–Organic Decomposition: Structure and Electrochemical Study for Application in Lithium-Ion Batteries

**DOI:** 10.3390/ma19132825

**Published:** 2026-07-02

**Authors:** Francisca Luco, Benjamín Silva, Andrés Ibáñez, Arianne Maine, Andrés Espinosa, Fabian Dietrich, Judit G. Lisoni, Víctor M. Fuenzalida, Rodrigo Espinoza, Marcos Flores

**Affiliations:** 1 Laboratory of Surfaces and Nanomaterials, Department of Physics, Faculty of Physical and Mathematical Sciences, Universidad de Chile, Santiago 8370448, Chile; benjasilva2014@gmail.com (B.S.); aibanez@dfi.uchile.cl (A.I.); arianne.maine@usach.cl (A.M.); aseczone@gmail.com (A.E.); vfuenzal@ing.uchile.cl (V.M.F.); 2 Laboratory of Multifunctional Advanced Materials, Department of Chemical Engineering, Biotechnology and Materials, Faculty of Physical and Mathematical Sciences, Universidad de Chile, Santiago 8370456, Chile; roespino@ing.uchile.cl; 3 Department of Material Chemistry (SMAT-C), Faculty of Chemistry and Biology, Universidad de Santiago de Chile, Santiago 9170022, Chile; 4 Department of Physics Science, Universidad de La Frontera, Temuco 4811230, Chile; fabian.dietrich@ufrontera.cl; 5 Institute of Physical and Mathematical Sciences, Sciences Faculty, Universidad Austral de Chile, Valdivia 5090000, Chile; judit.lisoni@uach.cl

**Keywords:** nanostructured thin films, LiNi_0.5_Mn_1.5_O_4_, lithium-ion batteries, metal–organic decomposition, 5 V cathode, binder-free cathode

## Abstract

The high-voltage spinel ${\mathrm{LiNi}}_{0.5}{\mathrm{Mn}}_{1.5}O_{4}$ (LNMO) is a promising cobalt-free cathode material for lithium-ion batteries, yet its integration as a binder-free thin film on metallic current collectors via simple solution routes remains underexplored. Here, LNMO films were synthesized on 304 stainless steel (SS304) by metal–organic decomposition (MOD) from metal–acetate precursors in ethanol, followed by spin-coating and annealing at 500, 600, and 700 °C under flowing $O_{2}$. The films were characterized by XRD, FESEM–FIB cross-sectioning, EDS, and XPS, and tested as binder-free cathodes by cyclic voltammetry and galvanostatic charge/discharge. All samples are dense, approximately 1.9 μm thick, and crystallize in the disordered spinel phase. The LNMO crystallite size increases from 21.9 to 43.8 nm between 500 and 700 °C, while the grain size also shows a temperature dependence, increasing the average size from 25 up to 56 nm in diameter. XPS confirms ${\mathrm{Mn}}^{4+}$ as the dominant manganese surface species (45–49%) across all samples. The films deliver reversible discharge capacities of 92, 92, and 70 mAh $g^{-1}$ at 0.1 C for LNMO500, LNMO600, and LNMO700, respectively, with well-defined ${\mathrm{Ni}}^{2+}{\mathrm{/Ni}}^{3+}$ and ${\mathrm{Ni}}^{3+}{\mathrm{/Ni}}^{4+}$ redox peaks at 4.7 and 4.8 V. DFT calculations independently predict a voltage plateau at ∼4.7 V for 0.2≤x≤1, in agreement with the experimental profiles. These findings establish MOD as a viable, vacuum-free route to the synthesis of nanostructured LNMO cathodes.

## 1. Introduction

The increasing demand for high-energy-density batteries in advanced electronic devices has intensified the need for sustainable materials synthesized via environmentally benign routes. Among the transition metal oxide materials, the spinel compound ${\mathrm{LiNi}}_{0.5}{\mathrm{Mn}}_{1.5}O_{4}$ (LNMO) stands out as one of the most promising candidates. It exhibits notable advantages, including a high operating voltage, ∼4.7 V vs. Li/Li^+^, a high theoretical energy density of 650 Wh ${\mathrm{kg}}^{-1}$, three-dimensional lithium-ion diffusion pathways, and enhanced safety characteristics [1]. Furthermore, its cobalt-free composition offers an eco-friendly and cost-effective alternative to conventional cobalt-based cathode chemistries.

Despite these advantageous attributes, bulk LNMO exhibits limited cycling stability under high-power regimes, primarily due to a rapid degradation process. This deterioration is particularly acute in conventional liquid electrolytes, where structural and chemical instabilities arising from Ni/Mn ratio fluctuations and the presence of ${\mathrm{Mn}}^{3+}$ ions induce severe interfacial reactions [2]. Under high-voltage conditions, ${\mathrm{Mn}}^{3+}$ undergoes disproportionation, leading to ${\mathrm{Mn}}^{2+}$ dissolution into the electrolyte, subsequent formation of the cathode–electrolyte interphase (CEI), and accelerated capacity fade [3]. This inherent incompatibility with the available liquid electrolyte systems has prompted the investigation of LNMO within solid-state configurations, notably thin-film lithium-ion batteries (TFLIBs), where the absence of liquid components circumvents these degradation mechanisms.

The electrochemical performance of the LNMO is strongly influenced by its crystalline structure. This material can crystallize in two different space groups: an ordered simple cubic structure (${\mathrm{P4}}_{3}$32), and a disordered face-centered cubic spinel (Fd$\bar{3}$m) [4,5,6]. In the ordered polymorph, Ni cations occupy specific 4b sites, whereas in the disordered phase, Ni distribution is randomized across 16c sites. This degree of cation ordering, in conjunction with particle morphology, significantly impacts cyclability and rate capability.

Thin-film cathode architectures have garnered significant attention for electrochemical energy storage applications owing to their high surface area, enhanced charge transfer kinetics, and compositional flexibility [7]. Spinel LNMO emerges as a particularly attractive material for TFLIBs and related solid-state devices [8], as the solid-state environment inherently suppresses the manganese dissolution prevalent in liquid electrolytes. Current fabrication methodologies for LNMO thin films, however, predominantly rely on physical vapor deposition techniques such as magnetron sputtering—processes that are often cost-prohibitive and typically require post-deposition thermal treatment to attain desired crystallinity [9]. While alternative approaches, including electrostatic spray deposition, have been demonstrated [10,11], the development of simpler, scalable synthesis strategies remains a critical need.

LNMO is conventionally synthesized as a bulk powder with micrometric particle dimensions employing techniques such as hydrothermal [12], sol-gel [13], electrospinning [14], pyrolysis by aerosol spraying [15], and co-precipitation [16]. Although some of these can be used to synthesize thin films, metal–organic decomposition (MOD) represents a promising solution-based route for producing stoichiometrically precise thin films. This methodology is cost-efficient, reproducible, environmentally sound, and suitable for large-area substrate coating without requiring vacuum infrastructure. The MOD process entails spin-coating a precursor solution containing metal–organic compounds onto a substrate, followed by thermal annealing to decompose the organometallic complexes into the desired oxide phase. Critical parameters—including precursor chemistry, solvent selection, spin-coating conditions, annealing temperature profile, dwell time, and atmosphere—exert profound influence on the film’s microstructure, chemical composition, and resultant electrochemical behavior [17,18]. The versatility of MOD has been validated through the synthesis of various functional oxides, including ${\mathrm{YMnO}}_{3}$ [19], gadolinium iron garnet (GdIG) [20], and conductive inks for printed electronics [21].

For electrochemical applications, precise control over the oxidation state of transition metals is paramount. Kim and colleagues highlighted that annealing in an oxygen-containing atmosphere is essential for achieving enhanced crystallinity and stabilizing the manganese oxidation state [22]. The presence of oxygen during thermal processing promotes the formation of Mn in oxidation states favorable for the development of a stable spinel structure.

In this study, LNMO thin films were synthesized on 304 stainless steel (SS304) substrates using metal–organic decomposition at temperatures of 500, 600, and 700 °C under a flowing oxygen atmosphere. To the best of our knowledge, this constitutes the first report of MOD-derived LNMO films used directly as binder-free and conductive additive-free cathodes in lithium-ion batteries.

## 2. Materials and Methods

### 2.1. Materials

Lithium acetate (LiCH_3_COO) (≥99.95%), nickel(II) acetate tetrahydrate (Ni(CH_3_COO)_2_·4 H_2_O) (≥99.0%), and manganese(II) acetate tetrahydrate (Mn(CH_3_COO)_2_·4 H_2_O) (≥99.0%) were purchased from Sigma-Aldrich (St. Louis, MO, USA). Lithium chips (≥99.95%) were acquired from Gelon Lib Group (Linyi, China), and absolute ethanol for HPLC was acquired from Scharlau (Barcelona, Spain).

### 2.2. Synthesis via Metal–Organic Decomposition

**Figure 1:**
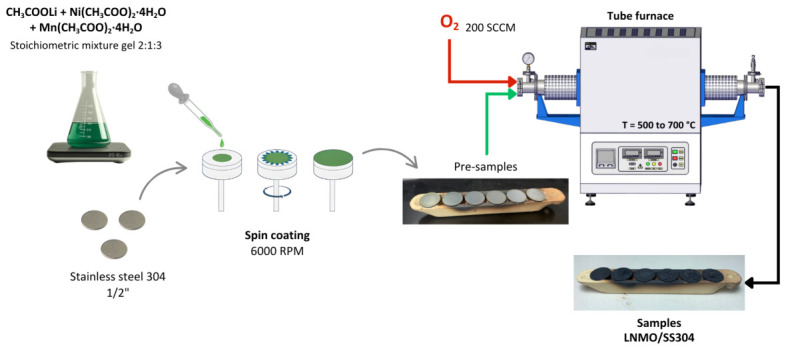
Scheme of the MOD methodology to synthesize the LNMO films.

The LNMO thin films were synthesized by employing a metal–organic decomposition (MOD) protocol, as shown schematically in Figure 1. The precursor solutions were formulated as stoichiometric mixtures of metal acetate compounds dissolved in absolute ethanol. The synthesis protocol initiated with a 4 h stirring phase at ambient temperature to ensure complete precursor dissolution and molecular-scale homogenization.

Following initial dissolution, the solution was progressively heated to 65 °C under continuous agitation to facilitate solvent evaporation and promote gelation through controlled polycondensation reactions. The resulting viscous gel exhibited optimal rheological properties for thin-film deposition.

Circular SS304 substrates (13 mm in diameter and 0.5 mm in thickness) were used as both structural supports and integrated current collectors. The selection of stainless steel over conventional carbon-coated aluminum current collectors was dictated by thermodynamic considerations, as the synthesis temperatures (500–700 °C) substantially exceed the melting point of aluminum.

Uniform film deposition was achieved via spin coating at 6000 RPM for 30 s, ensuring homogeneous coverage across the entire substrate surface. The as-deposited gel precursors underwent thermal processing in a tubular furnace under controlled oxygen atmosphere (200 SCCM $O_{2}$ flow), utilizing alumina crucibles to prevent contamination. The thermal profile consisted of (i) rapid heating at 25 °C/min to target temperatures (500, 600, or 700 °C), (ii) a 1 h isothermal dwell to facilitate complete precursor decomposition and crystalline phase formation, and (iii) controlled cooling at 5 °C/min to ambient temperature to minimize thermal stress and preserve structural integrity. Finally, the mass of each sample was determined using a Radwag microbalance (model AS 82/220.R2). The recorded value was subtracted from the mass of the corresponding SS304 substrates, thereby yielding the net mass of the synthesized cathodic material. The average mass of cathodic material was 2.36±0.05 mg.

### 2.3. Film Characterization

The LNMO films obtained were structurally characterized by X-ray diffraction (XRD) using a Bruker D8 X-ray diffractometer (Karlsruhe, Germany. Cu K$\alpha_{1}$, λ = 1.5604 Å, operating at 40 kV and 30 mA) at room temperature. DIFFRAC.EVA software, version 15.0.0.0 was used for phase identification, and Rietveld refinement was performed by DIFFRAC.TOPAS version 4.2 Bruker AXS software.

The morphology was examined using Field Emission Scanning Electron Microscopy (FESEM) with an Inlens Energy-Selective Backscatter (ESB) Detector, in combination with Focused Ion Beam (FIB) techniques. This was conducted on a Zeiss Auriga Crossbeam (Oberkochen, Germany) at accelerating voltages ranging from 1 to 3 kV. To improve the FIB cross-section, platinum was strategically deposited onto the samples through sputtering, achieving a thickness of less than 5 nm. The FIB erosion measurement was conducted at an angle of 54 degrees. The chemical composition of the films was determined by energy-dispersive X-ray spectroscopy with an Oxford Instruments X-act 10 mm^2^ detector (129 eV resolution, GSR AZtec Energy software, version 6.0SP1) in a Zeiss EVO MA10 SEM. ImageJ, Version 1.54p, was utilized to analyze the micrographs. To determine of the average particle size and the average film thickness, a total of 300 particles were quantified from images captured from a top-down perspective, while 100 independent thickness measurements were recorded along the cross-sections.

The Raman measurements were performed on a Witec Alpha300 RA confocal Raman microscope (Ulm, Germany). An Ar laser with an excitation wavelength of 532 nm, a 100× microscope objective with a numerical aperture of 0.75, and an electrically cooled CCD camera were used in all measurements. The laser power on the samples was <1.3 mW, the spectral resolution was 5 cm^−1^, and 100 scans per second were performed. The spectra were recorded within the 100–1000 cm^−1^ region. Spectra were acquired from all films using a confocal configuration with a working distance of 0.31 mm and spatial resolution of 1 μm.

To evaluate the chemical states of the surface atomic species, X-ray photoelectron spectroscopy (XPS) data were acquired. Measurements were conducted using a SPECS XPS spectrometer (Berlin, Germany) with a PHOIBOS 150 hemispherical electron energy analyzer and a 1D-DLD detector. The X-ray source, consisting of an Al anode (hν=1486.6 eV), provided unfiltered Kα radiation. The main spectrometer chamber pressure was maintained at approximately $10^{-7}$ Pa during data acquisition [23]. The binding energy (BE) scale was calibrated using the C1s peak of the carbon support, set to 284.8 eV. Surface atomic ratios were computed by integrating peak areas normalized by the acquisition parameters, atomic sensitivity factors (ASFs), and transmission functions provided by the manufacturer.

### 2.4. Electrochemical Cell Assembly and Performance Evaluation

Following cathode fabrication, all sample handling and cell assembly procedures were conducted within an argon-filled glovebox maintaining stringent atmospheric controls (<1 ppm H_2_O and O_2_). Two-electrode Swagelok-type half-cells were configured with LNMO films as working electrodes and lithium metal foil as both the counter and reference electrodes.

The electrochemical cells incorporated a standardized active area of 1.3 cm^2^ for all cathodes. The electrolyte formulation consisted of 1 M lithium hexafluorophosphate (LiPF_6_) dissolved in a 1:1 (*v*/*v*) binary solvent mixture of ethylene carbonate (EC) and dimethyl carbonate (DMC). A Whatman GF/D glass fiber separator, pre-saturated with the liquid electrolyte, provided ionic conduction while preventing electrical contact between electrodes.

Electrochemical characterization was performed using a BioLogic BCS-805 battery testing system (Seyssinet-Pariset, France) controlled via BT-Lab V1.79 software. Cyclic voltammetry measurements were acquired across a voltage window of 3.0–5.0 V versus Li/Li^+^. In addition, galvanostatic charge/discharge profiles at increasing current rates, 0.1 C, 0.2 C, 0.5 C, 1 C and 2 C, were acquired to evaluate high-power performance.

### 2.5. DFT Simulations

First-principles calculations were performed using density functional theory (DFT) as implemented in the VASP 6.3.2 [24,25]. The interaction between valence electrons and ionic cores was described using the projector augmented-wave (PAW) method [26], while the electronic wavefunctions were expanded in a plane-wave basis set with a kinetic energy cutoff of 520 eV. Exchange-correlation effects were treated within the generalized gradient approximation (GGA) using the Perdew–Burke–Ernzerhof (PBE) functional [27]. To account for the localized nature of the transition-metal d electrons, the DFT+U method in the Dudarev formulation [28] was employed with effective Hubbard parameters $U_{\mathrm{eff}}$ = 3.9 eV for Mn and $U_{\mathrm{eff}}$ = 6.2 eV for Ni. To account the magnetic properties, spin-polarized calculations were performed. Structural optimizations were carried out using a Γ-centered k-point mesh generated from a reciprocal-space spacing of 0.22 Å^−1^. Both lattice parameters and atomic positions were relaxed until the residual forces on all atoms were below 0.02 eV Å^−1^, while the electronic self-consistency criterion was set to 10^−5^ eV. The Brillouin-zone integrations during geometry optimization employed Gaussian smearing with a width of 0.05 eV. Following structural relaxation, single-point electronic energy calculations were performed using the optimized geometries. For these calculations, a denser Γ-centered k-point mesh corresponding to a reciprocal-space spacing of 0.16 Å^−1^ was used together with the tetrahedron method including Blöchl corrections for Brillouin-zone integration. The electronic self-consistency criterion was tightened to 10^−6^ eV.

**Figure 2:**
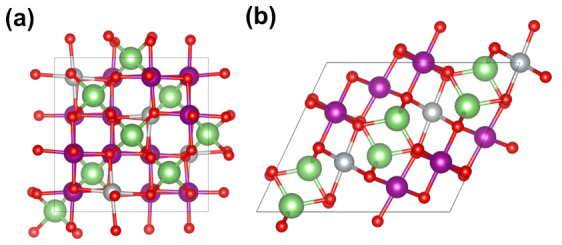
Unit cells of LMNO: (**a**) P4_3_32, (**b**) Fd$\bar{3}$m. Color code: Li—green, Ni—grey, Mn—violet, O—red.

Experimental crystal structures of LiNi_0.5_Mn_1.5_O_4_ were obtained from The Materials Project page [29] for the ordered phase (space group P4_3_32, [30]) and the desordered phase (adapted from LiMn_2_O_4_, space group Fd$\bar{3}$m). The first one contains 8 Li, 12 Mn, 4 Ni and 32 O atoms in the unit cell, the latter one 6 Li, 8 Mn, 3 Ni and 24 O atoms. Both are shown in Figure 2.

The structures were optimized using the above-mentioned DFT methods, resulting in only a slight change in the lattice parameters. For the P4_3_32 phase, the 8 Li atoms in the unit cell can be conveniently removed to obtain concentrations for Li_x_Ni_0.5_Mn_1.5_O_4_ with x = 0, 1/8, 1/4, 3/8, 1/2, 5/8, 3/4, 7/8 or 1, while for the Fd$\bar{3}$m phase, x = 0, 1/6, 1/3, 1/2, 2/3, 5/6, or 1 are available.

The electrostatic potential *U* can be obtained according to the expression:

(1) $$U=-\frac{E\left({\mathrm{Li}}_{x}{\mathrm{Ni}}_{0.5}{\mathrm{Mn}}_{1.5}O_{4}\right)-E\left({\mathrm{Li}}_{x^{\prime }}{\mathrm{Ni}}_{0.5}{\mathrm{Mn}}_{1.5}O_{4}\right)-\left(x-x^{\prime }\right)E\left(\mathrm{Li}\right)}{\left(x-x^{\prime })|e\right|},\mathrm{with}\;x-x^{\prime }=\frac{1}{8}\;\mathrm{or}\;\frac{1}{6}$$

where *E* is the total (DFT) energy of the compound in parentheses, *x* is the Li concentration, and *e* is the elemental charge. The positions of the Li atoms for 0<x<1 are equivalent. However, in the case that more than 1 atom is inserted, permutations of the positions have to be considered, yielding n8 or n6 options, respectively. Considering those permutations (and their ponderation *n*), an average electrostatic potential for a certain Li concentration can be calculated:

(2) $$\bar{U}=\frac{\sum_{i}n_{i}U_{i}}{\sum_{i}n_{i}}$$

For comparison with the experimental data, the different lithium concentrations are related to the theoretical capacity *C*, using the molar mass *M*:

(3) $$C=\frac{x|e|}{M}$$

## 3. Results

### 3.1. Structural Characterization of LNMO Films

**Figure 3:**
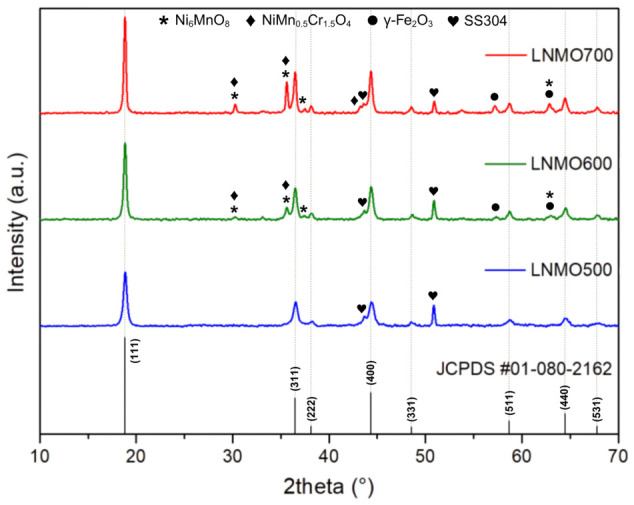
X-ray diffraction patterns of LNMO thin films synthesized at 500, 600, and 700 °C on SS304 substrates. Diffraction peaks are indexed to the ordered spinel phase (${\mathrm{P4}}_{3}$32, JCPDS #01-080-2162). Secondary phases identified include: (*) ${\mathrm{Ni}}_{6}{\mathrm{MnO}}_{8}$, (⧫) ${\mathrm{NiMn}}_{0.5}{\mathrm{Cr}}_{1.5}O_{4}$, and (•) γ${\mathrm{-Fe}}_{2}O_{3}$. Substrate peaks from SS304 are marked with (♥).

Figure 3 shows the XRD patterns corresponding to the LNMO thin-film samples prepared by the MOD method. The diffractograms are presented after background subtraction to minimize fluorescence interference caused by the iron in the stainless-steel substrate. The diffractograms for all synthesized samples were indexed against the JCPDS card #01-080-2162, corresponding to the LiNi_0.5_Mn_1.5_O_4_ spinel phase. It should be noted that crystallographic texture, common in thin-film deposition, results in relative peak intensities that differ from those of randomly oriented powder standards. Characteristic diffraction peaks of the ${\mathrm{LiNi}}_{0.5}{\mathrm{Mn}}_{1.5}O_{4}$ phase were identified at 2θ angles of 18.8°, 36.4°, 38.1°, 44.3°, 48.5°, 58.6°, 64.4°, and 67.8°.

An enhancement in peak intensity is observed for the (111) and (531) planes (2θ = 18.8° and 67.8°, respectively) with increasing calcination temperature, indicating improved crystallinity at higher synthesis temperatures. For the film synthesized at 500 °C, the diffraction pattern reveals the presence of the target LNMO phase alongside reflections originating from the stainless-steel substrate at 2θ = 43.6° and 50.8°, corresponding to the (111) and (200) planes, respectively. The detection of the substrate signals across all samples is attributed to the penetration depth of the X-ray beam, which interacts with the underlying micrometer-thick stainless steel. In samples prepared at 600 °C and 700 °C, additional diffraction peaks emerge at 2θ = 30.2°, 35.6°, and 37.5°, which are assigned to the cubic ${\mathrm{Ni}}_{6}{\mathrm{MnO}}_{8}$ phase (planes (220), (311), and (222)). The intensity of these secondary phase peaks exhibits a positive correlation with synthesis temperature, suggesting that elevated temperatures facilitate their formation. Furthermore, in the sample calcined at 700 °C, the appearance of reflections at 2θ = 35.6° and 43.2° indicates the formation of a ${\mathrm{NiMn}}_{0.5}{\mathrm{Cr}}_{1.5}O_{4}$ spinel phase.

Additional peaks observed at 2θ = 57.3° and 62.8° are indicative of γ${\mathrm{-Fe}}_{2}O_{3}$ (planes (511) and (440), respectively). The formation of iron oxide species is consistent with the oxidation of stainless steel when exposed to high temperatures under an oxidizing atmosphere [31]. The absence of these peaks in the 500 °C sample suggests that their formation is thermally activated.

**Figure A1:**
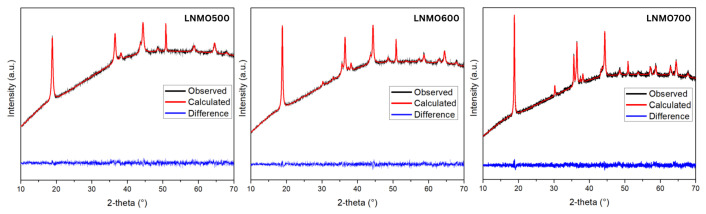
Rietveld refinement from the raw XRD data of LNMO samples synthesized at different temperatures.

Rietveld refinement was performed on the raw diffraction data; the corresponding fits are presented in Figure A1, and the quantitative phase analysis is summarized in Table 1. The refinement results indicate that secondary phases are present in minor proportions in the film synthesized at 600 °C, with their abundance increasing at higher temperatures. Identifying these secondary phases is important for understanding the high-temperature limits of LNMO deposition via MOD. The formation of the ${\mathrm{Ni}}_{6}{\mathrm{MnO}}_{8}$ phase is attributed to the volatilization of lithium during the MOD process, creating sites Li-deficient environment that forces the segregation of Ni and Mn into stable oxide complexes. The emergence of the Cr-substituted spinel ${\mathrm{NiMn}}_{0.5}{\mathrm{Cr}}_{1.5}O_{4}$ provides direct evidence of solid-state interdiffusion across the film–substrate interface. At high temperatures, ${\mathrm{Cr}}^{3+}$ ions from the stainless-steel substrate migrate into the film due to their high octahedral site stabilization energy, partially replacing Mn ions in the spinel lattice.

The lattice parameter of the LNMO phase remains invariant with respect to the synthesis temperature. In contrast, both crystallite size and average particle size exhibit a clear dependence on thermal treatment. The comparable values obtained for crystallite and particle dimensions suggest that the LNMO particles are predominantly monocrystalline in nature.

Raman spectroscopy was employed to complement the structural information obtained from XRD analysis. The resulting spectra are consistent with the disordered Fd$\bar{3}$m space group [32], with confocal measurements indicating that this phase predominates in the film and close to the outermost surface. Additional confocal analysis (not included here) revealed spectral features corresponding to the secondary phases identified by XRD, confirming that these components are localized primarily at the interface between the LNMO films and the SS304 substrate.

**Figure 4:**
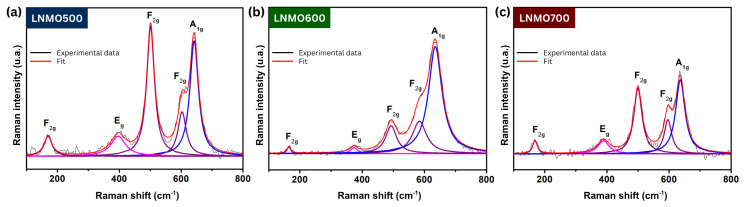
Raman spectra of LNMO films synthesized at (**a**) 500 °C, (**b**) 600 °C, and (**c**) 700 °C. Characteristic vibrational modes are labeled: (Mn–O) at 602 and $641{\mathrm{cm}}^{-1}$, (Ni–O) at 395 and $501{\mathrm{cm}}^{-1}$, and (Li–O) at $167{\mathrm{cm}}^{-1}$. The spectra are normalized and offset for clarity.

Figure 4 presents the averaged Raman spectra and their corresponding deconvolution, providing a detailed assignment of vibrational bands associated with the transition metal oxide components. All samples exhibit characteristic peaks at approximately 602 ($F_{2g}$) and $641{\mathrm{cm}}^{-1}$ ($A_{1g}$), which are attributed to the symmetric stretching mode of Mn−O bonds in ${\mathrm{Mn}}^{4+}$-containing octahedra. Two additional prominent bands observed at approximately 395 ($E_{g}$) and $501{\mathrm{cm}}^{-1}$ ($F_{2g}$) corresponding to Ni−O stretching vibrations arising from ${\mathrm{Ni}}^{4+}$ octahedra within the spinel framework. The intensity of the peak at approximately $167{\mathrm{cm}}^{-1}$ ($F_{2g}$), associated with lattice vibrations involving Li−O bonds, serves as a sensitive indicator of the degree of cationic ordering in the LNMO structure [33].

Spectral analysis reveals systematic variations in peak positions, intensities, and full-widths at half-maximum (FWHMs) across samples synthesized at different temperatures, reflecting changes in crystallinity, stoichiometry, and potential cation redistribution. The relative intensity ratios between ordered and disordered phase signatures provide quantitative insights into the temperature-dependent evolution of the LNMO structure and its interfacial characteristics.

### 3.2. Morphological and Elemental Analysis

**Figure 5:**
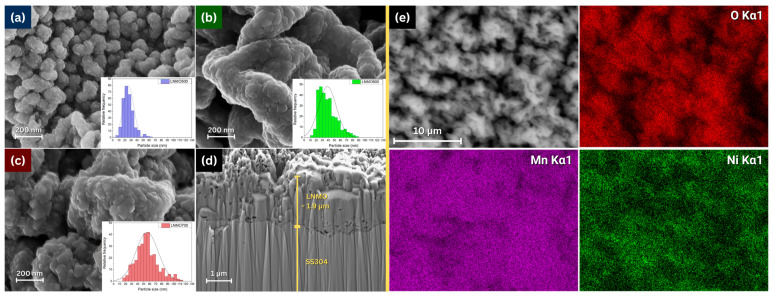
FESEM images and particle size distribution histograms of (**a**) LNMO500, (**b**) LNMO600, (**c**) LNMO700, (**d**) FIB-SEM cross-sectional image of LNMO700, and (**e**) EDS mapping.

Figure 5 presents FESEM micrographs representative of each synthesized sample. The microstructural analysis reveals the formation of homogeneous films on the SS304 substrates, composed primarily of octahedral nanostructures whose dimensions exhibit a clear dependence on synthesis temperature. Particle size distribution histograms (insets in the same figure) demonstrate that elevated synthesis temperatures yield films with increased grain sizes, a trend quantitatively corroborated by the average values summarized in Table 1.

Cross-sectional analysis of the LNMO film synthesized at 700 °C, as shown in Figure 5d, reveals the uniformity of the films with a nominal thickness of 1.9±0.3 μm, which is representative of all samples investigated, indicating that film thickness remains invariant with synthesis temperature. The interface between the LNMO layer and the SS304 substrate is well-defined. The absence of contrasting features (e.g., white or dark spots) along the film suggests homogeneous material distribution without significant phase segregation, consistent with the coexistence of secondary phases within the LNMO matrix. The vertical striations observed in the cross-section are artifacts resulting from the focused gallium ion beam milling process.

The LNMO700 film exhibits a moderate degree of porosity; however, no evidence of material infiltration or embedding within the porous structure is observed. Furthermore, microstructural analysis provides no indication of crack formation induced by oxidative conditions at the substrate interface, which would otherwise facilitate liquid electrolyte penetration and compromise electrochemical stability.

Energy-dispersive X-ray spectroscopy (EDS) spectra were acquired from all samples. Compositional analysis was performed via both mapping and spot analysis techniques, the latter employing nine distinct points to derive average elemental concentrations. The characteristic elements of the LNMO phase—Mn, Ni, and O—are consistently identified across all samples, see Table 2. The lithium content could not be quantified due to the detection limitations of the EDS system. Although Fe and Cr signals from the substrate are detectable owing to the lateral and depth resolution of the technique, these elements were excluded from the quantitative analysis of the film composition.

Elemental mapping, as shown in Figure 5e, confirms the homogeneous distribution of all the constituent elements within the film. Concentration profiles indicate preferential accumulation of the LNMO-related elements (Mn, Ni, O) toward the film surface, while substrate-derived elements (Fe, Cr) remain predominantly concentrated in the underlying regions.

To further investigate interdiffusion phenomena, linear cross-sectional FIB-EDS mapping was performed on LNMO700 samples. The analysis reveals measurable migration of Fe and Cr species from the SS304 substrate into the interfacial region of the film, consistent with the formation of secondary phases containing these elements (as identified by XRD). This interdiffusion, however, appears confined to the interface, with no significant migration reaching the film surface. These observations confirm that secondary phases are uniformly distributed at the LNMO-SS304 interface rather than throughout the bulk film.

**Figure 6:**
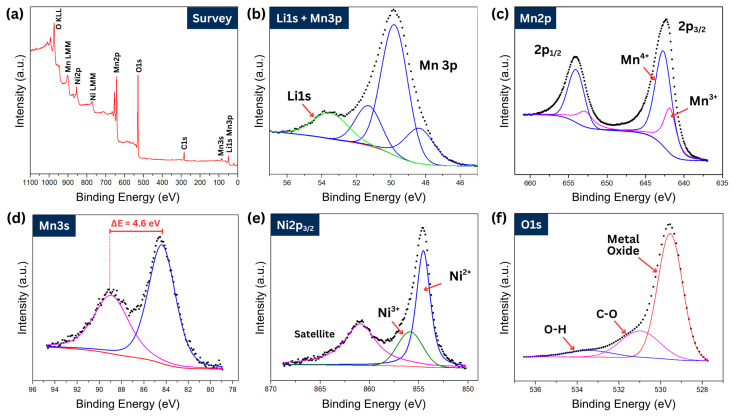
Representative XPS spectra of MOD-synthesized LNMO films: (**a**) survey and (**b**–**f**) narrow spectra for each atomic species.

X-ray photoelectron spectroscopy (XPS) was employed to characterize the surface elemental composition and the constituent chemical and oxidation states of the LNMO films. As a surface-sensitive technique with an analysis depth of approximately 5–10 nm, XPS provides high-resolution data regarding the chemical environment within the uppermost atomic layers. The survey and high-resolution spectra. Figure 6. confirm the presence of Li, Ni, Mn, and O as the primary constituents in all films, and the atomic percent of the identified species are included in Table A1. In particular, the absence of Fe or Cr signals—characteristic of the SS304 substrate—indicates a continuous film morphology devoid of pinholes or delamination. Furthermore, the spectral features remain consistent across samples synthesized at different temperatures, with binding energies (BEs) falling within identical ranges. This uniformity suggests that while synthesis temperature may influence bulk structural properties, the surface stoichiometry remains largely homogeneous within the XPS detection limits.

The following section presents a detailed analysis of the narrow signals. First, the Li1s core-level spectrum overlaps with Mn3p transitions, as shown in Figure 6b, a feature characteristic of Li-O bonding in spinel oxide frameworks [34] at a binding energy close to 53.7 eV. The stable Li1s peak intensity across the temperature series indicates uniform lithium distribution and minimal surface lithium loss during thermal processing, consistent with previous reports by Wang et al. [35] and Bhuvaneswari et al. [36]. Second, the manganese oxidation state is obtained from the Mn2p signal, as shown in Figure 6c, displaying a characteristic doublet with ${\mathrm{Mn2p}}_{3/2}$ and ${\mathrm{Mn2p}}_{1/2}$ at binding energy of 642.0 eV and 653.4 eV, atributed to ${\mathrm{Mn}}^{3+}$, and a second doublet at binding energy of 642.6 eV and 654.2 eV, atributed to ${\mathrm{Mn}}^{4+}$ [3]. The ${\mathrm{Mn2p}}_{3/2}$ binding energy corresponds predominantly to ${\mathrm{Mn}}^{4+}$ species, which are essential for maintaining structural stability and electrochemical reversibility during lithiation/delithiation. The ΔBE(${\mathrm{Mn2p}}_{1/2}-{\mathrm{Mn2p}}_{3/2}$) value of 11.6 eV further validates the ${\mathrm{Mn}}^{4+}$ state, typically ≈ 11.7 eV for ${\mathrm{MnO}}_{2}$, distinguishing it from ${\mathrm{Mn}}^{2+}$, in MnO ≈ 11.0 eV, and ${\mathrm{Mn}}^{3+}$, in ${\mathrm{Mn}}_{2}O_{3}$, ≈ 11.3 eV. For completeness, the Mn3s signal was studied, as shown in Figure 6d. It presents two characteristic peaks with a binding energy separation ΔBE of 4.6 eV across all samples, confirming that the surfaces are dominated by ${\mathrm{Mn}}^{4+}$ regardless of the synthesis temperature. Third, the ${\mathrm{Ni2p}}_{3/2}$ signal reveal a mixed-valence environment comprising ${\mathrm{Ni}}^{2+}$ and ${\mathrm{Ni}}^{3+}$ species, as shown in Figure 6e, at binding energies of 854.5 eV and 855.8 eV, respectively. Here, it is dominated by the ${\mathrm{Ni}}^{2+}$ oxidation state. Finally, the O1s signal identifies multiple chemical environments, as shown in Figure 6f, corresponding to metal–oxygen bonds alongside adventitious surface species such as hydroxyls and carbonates. The binding energies and corresponding chemical assignments are summarized in Table 3, and the relative area percentages derived from peak fitting for each bond or oxidation state are also provided.

Based on the EDS and XPS data, as shown in Table 2 and Table A1, respectively, the Mn:Ni ratio was determined for each sample, reported in Table 4. Based on the stoichiometric balance of the ideal ${\mathrm{LiMn}}_{1.5}{\mathrm{Ni}}_{0.5}O_{4}$ phase, the theoretical Mn:Ni ratio is 3:1. Conversely, the surface-sensitive XPS measurements revealed a Mn:Ni ratio close to 3.1:1. This indicates that the pure LNMO phase is predominantly located near the surface, a finding highly consistent with the Raman spectroscopy observations. Regarding the ${\mathrm{Mn}}^{3+}{\mathrm{/Mn}}^{4+}$ ratio, from XPS, it ranged from 1:2.6 to 1:4.5, indicating the formation of a disordered phase LNMO spinel structure, possibly with oxygen vacancies. This is consistent with the disordered phase identification from Raman measurements. XPS analysis also allows for the evaluation of the lithium content by extracting the relative peak areas from the combined Li1s+Mn3p signals. The atomic concentrations were computed utilizing the atomic sensitivity factors (ASFs) from the MultiPak software, version 9.4.0.7 following the ussual methodology [23]. The resulting Li:Mn ratio is close to 1:1, whereas the theoretical value expected for the stoichiometric phase is 1:1.5.

### 3.3. Electrochemical Study

**Figure 7:**
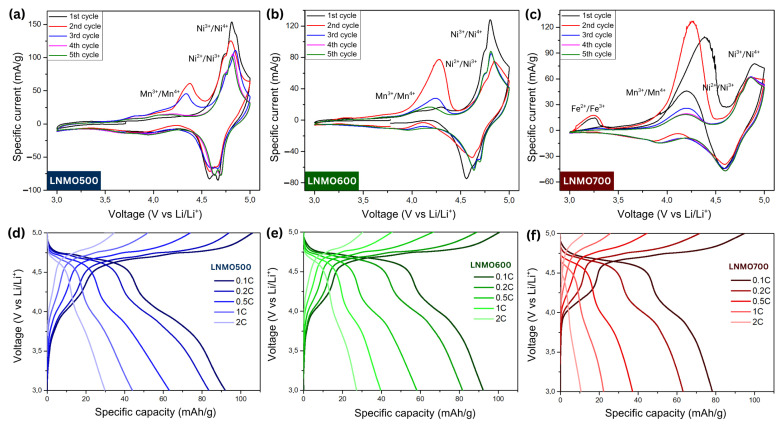
Comparison of electrochemical performance of LNMO films: (**a**–**c**) cyclic voltammetry curves’ initial five cycles, and (**d**–**f**) charge/discharge profiles at different rates.

In this section, we present the electrochemical study of LNMO films employed directly as cathodes in half-cell configurations. Figure 7a–c display the initial five cycles of the cyclic voltammetry measurements at a scan rate of 0.1 mV/s to evaluate redox reversibility, lithium-ion diffusion kinetics, and polarization behavior.

We identified the redox peaks corresponding to the two-stage ${\mathrm{Ni}}^{2+}{\mathrm{/Ni}}^{3+}$ and ${\mathrm{Ni}}^{3+}{\mathrm{/Ni}}^{4+}$ couples at approximately 4.7 V and 4.8 V, respectively. Additionally, less intense peaks associated with the ${\mathrm{Mn}}^{3+}{\mathrm{/Mn}}^{4+}$ redox couple were identified at 4.1 V. It is observed that the peak positions for both Ni and Mn vary very little in voltage, but a clear evolution of the current intensity is seen between each cycle. After the first few cycles, a decrease in the current peaks of nickel oxidation was observed; in contrast, an increase in the current peaks of manganese oxidation was observed, which stabilized from the third cycle onward. This behavior can be attributed to the formation and stabilization of the CEI layer. The presence of ${\mathrm{Mn}}^{4+}$ as the dominant surface species is confirmed, though the CV reveals residual electrochemical activity of ${\mathrm{Mn}}^{3+}$, consistent with HR-XPS analysis indicating minor ${\mathrm{Mn}}^{3+}$ surface content. This observation aligns with literature reports suggesting limited electrochemical activity of ${\mathrm{Mn}}^{4+}$ species [37].

The specific current magnitude exhibits a strong dependence on synthesis temperature, directly correlated with microstructural evolution. LNMO500 and LNMO600 samples display sharper redox peaks and higher current responses, indicative of enhanced lithium-ion diffusivity and electrochemical activity. In contrast, LNMO700 exhibits attenuated peak intensities and reduced specific currents, attributed to increased secondary phase content that diminishes electrochemically active material fraction. The ${\mathrm{Fe}}^{2+}{\mathrm{/Fe}}^{3+}$ redox for LNMO700 can be observed, but only for the first two cycles.

Figure 7d–f display charge/discharge profiles for the LNMO500, LNMO600, and LNMO700 samples. The measurements were conducted at 0.1 C, 0.2 C, 0.5 C, 1 C and 2 C in the range of 3.0–5.0 V at room temperature. All samples at different rates of charge/discharge show two voltage plateaus: one at approximately 4.0 V, attributed to the ${\mathrm{Mn}}^{3+}{\mathrm{/Mn}}^{4+}$ redox couple, and another at approximately 4.7 and 4.8 V, corresponding to the ${\mathrm{Ni}}^{2+}{\mathrm{/Ni}}^{3+}$ and ${\mathrm{Ni}}^{3+}{\mathrm{/Ni}}^{4+}$ couples, respectively. This chemical composition has been reported in the literature to favor lithium diffusion kinetics in electrochemical tests. The coexistence of ${\mathrm{Mn}}^{3+}$ and ${\mathrm{Mn}}^{4+}$ enhances the electronic conductivity of the film via small-polaron hopping [38]; while it is true that excessive ${\mathrm{Mn}}^{3+}$ can trigger Jahn–Teller distortions and ${\mathrm{Mn}}^{2+}$ dissolution via disproportionation, the literature shows that a moderate amount of ${\mathrm{Mn}}^{3+}$ under 25%, in disordered LNMO, actually stabilizes the structure during high-voltage cycling by suppressing the detrimental two-phase transition and promoting a smoother solid-solution mechanism during lithium extraction/insertion [3,39,40].

Galvanostatic charge/discharge profiles at 0.1 C reveal charge capacities of 106 mAh g^−1^ (LNMO500) and 101 mAh g^−1^ (LNMO600), with both cathodes delivering identical discharge capacities of 92 mAh g^−1^. This suggests that while charge capacity is influenced by synthesis temperature, discharge capacity remains comparable for intermediate temperatures. LNMO700 demonstrates reduced performance with charge and discharge capacities of 78 mAh g^−1^ and 70 mAh g^−1^, respectively, underscoring the importance of phase purity, crystallinity, and optimal particle size for high capacity retention. The reduction in capacity with increasing synthesis temperature is closely linked to the crystallization of secondary ${\mathrm{Ni}}_{6}{\mathrm{MnO}}_{8}$ and ${\mathrm{NiMn}}_{0.5}{\mathrm{Cr}}_{1.5}O_{4}$ impurity phases, as shown in Table 1. Consequently, these impurity phases decrease the volume fraction of the pure active LNMO phase. To avoid the formation of unwanted impurity phases, it is necessary to use lower deposition temperatures or the integration of diffusion barriers for higher temperatures, such as ${\mathrm{TiO}}_{2}$.

**Figure 8:**
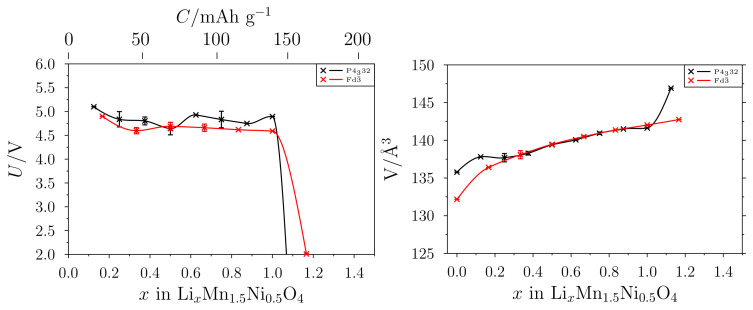
Voltage (**left**) and normalized volume of the unit cell (**right**) as a function of the Li concentration obtained from DFT calculations.

The simulated voltage curves as a function of the Li ion concentration are shown in Figure 8 left, where plateaus can be found for a concentration *x* between 0.2 and 1.0, for both investigated species, resulting in a voltage of about 4.7 V, representing well the experimental values. The volume of the optimized cells was normalized to one Li_x’_NiMn_3_O_6_ unit in order to directly compare both P4_3_32 and Fd$\bar{3}$m. As shown in Figure 8 right, the volume increases with the increasing amount of Li^+^ ions. These changes are small, revising the range of the plateaus and resulting in an increase from 137 Å^3^ to 142 Å^3^, which represents an increase of only 3.7%. In the case of P4_3_32, the absorption of a further Li^+^ ion results in a higher increase in volume. Together with the significant drop in voltage at this Li^+^ ion concentration, a structural reorganization can be concluded. For Fd$\bar{3}$m, the voltage also drops significantly at values beyond x=1. This means that lithiation with more than one Li per LMNO unit is unfavorable. The DFT simulations revealed an interesting behavior. Since we performed spin-polarized simulations, the magnetic moment of the metals could be monitored. For all studied cases, the resulting magnetic moments of the manganese are around 3$\mu_{B}$, which represents Mn^4+^, while nickel is either Ni^2+^ (2$\mu_{B}$) or Ni^3+^ (1–1.3$\mu_{B}$). For several Li^+^ ion concentrations, both magnetic state results compete in the simulation, with the lower-magnetic-moment state having the lower minimum energy. This means, the Ni^3+^ is more likely to be present.

Comparing the theoretical voltage curves with the experimental results, we observe that the voltage drop in the simulations occurs at capacities between 140 and 150 mAh g^−1^, which is more than fifty percent higher than the experimentally measured value. This discrepancy can be attributed to the simplified simulation model, which represents an ideal crystal at 0 K and therefore neglects entropic contributions. Furthermore, the simulations assume an infinitely slow lithium insertion process, corresponding to thermodynamic equilibrium conditions that differ from the finite-rate processes occurring in experiments. In particular, the last argument can explain the discrepancy, since the same experimental results show a strong dependency of charge rate on the extension of the plateau. Furthermore, in the experiment, a phase change at a Li^+^ ion concentration below x=1 may occur, which is not considered in the DFT simulation.

On the other hand, our DFT calculations model lithium intercalation from a vacuum. However, experimentally, at 4.7 V–4.9 V, the commercial electrolyte undergoes severe decomposition. This decomposition generates a CEI layer onto the LNMO film. Because the LNMO film has a high roughness, the actual surface area is larger, resulting in a thicker and more disordered CEI layer. This layer consumes current, lowering coulombic efficiency, and physically blocks lithium diffusion, reducing practical capacity. An approach to reducing CEI side effects is surface functionalization using PPABA molecules [41].

## 4. Conclusions

Spinel LNMO films were synthesized at 500 °C, 600 °C, and 700 °C, achieving thicknesses of less than 2 μm on stainless-steel substrates. The MOD method proved to be effective for the fabrication of nanostructured LNMO films without carbon black or binders. Reproducibility was demonstrated in all samples, manufacturing steps were reduced, and the synthesis temperature was lowered compared to traditional methods for the synthesis of LNMO powders and films using magnetron sputtering. The desired phase was obtained; however, secondary phases were detected as the synthesis temperature increased. The material synthesized at all these temperatures shows a favorable electrochemical response, with reversible discharge capacities of 92, 92, and 70 mAh $g^{-1}$ at 0.1 C for LNMO500, LNMO600, and LNMO700, respectively, and well-defined Ni redox couples at 4.7 and 4.8 V that match the voltage plateau predicted by DFT for 0.2≤x≤1. However, this capacity decreases as electrochemically inactive secondary phases appear, which identifies 500–600 °C as the optimal processing window for MOD-derived LNMO cathodes.

## Figures and Tables

**Table 1:** Structural parameters obtained from Rietveld refinement of LNMO films synthesized at different temperatures.

Sample	Phase Composition (%)			Crystal Parameters of LiNi_0.5_Mn_1.5_O_4_		
	NiMn_0.5_Cr_1.5_O_4_	Ni_6_MnO_8_	LiNi_0.5_Mn_1.5_O_4_	Lattice Parameter (Å)	Crystallite Size (nm)	Average Particle Size (nm)
LNMO500	–	–	100.00	8.168	21.9	25±9
LNMO600	0.74	1.86	97.40	8.170	31.3	38±15
LNMO700	7.65	3.64	88.71	8.170	43.8	56±18

**Table 2:** Overall average atomic composition with their standard deviations (SD) for each LNMO sample.

Element	LNMO500 (at.%)	LNMO600 (at.%)	LNMO700 (at.%)
O	44 ± 27	43 ± 28	56 ± 6
Mn	32 ± 17	28 ± 2	21 ± 9
Fe	10 ± 8	17 ± 20	14 ± 13
Ni	10 ± 5	8 ± 3	6 ± 3
Cr	4 ± 2	4 ± 4	2 ± 2
S	<1	<1	<1

**Table 3:** Surface chemistry of MOD-synthesized LNMO films determined by XPS: binding energies (BEs, in eV) and relative areas (%) of the fitted components for each signal.

Region	Component	LNMO500		LNMO600		LNMO700	
		BE (eV)	%	BE (eV)	%	BE (eV)	%
Li1s	Li–Mn–O	53.6	100	53.8	100	53.7	100
O1s	Mn–O/Ni–O	529.5	68	529.6	70	529.4	68
	C–O	531.0	25	531.0	23	530.8	24
	O–H	533.1	7	533.4	7	533.1	8
Mn2p_3/2_	Mn^3+^	642.0	10	642.0	17	641.9	13
	Mn^4+^	642.6	45	642.8	45	642.5	49
Ni2p_3/2_	Ni^2+^	854.5	42	854.5	40	854.3	39
	Ni^3+^	855.8	17	855.8	21	855.5	21

**Table 4:** Ni:Mn ratios and oxidation state distributions for LNMO samples derived from bulk-sensitive EDS and surface-sensitive XPS.

Sample	from EDS	from XPS		
	Ni:Mn	Ni:Mn	Ni^2+^:Ni^3+^	Mn^3+^:Mn^4+^
LNMO500	1:3.2	1:3.1	2.5:1	1:4.5
LNMO600	1:3.5	1:3.0	1.9:1	1:2.6
LNMO700	1:3.5	1:3.1	1.8:1	1:3.8

**Figure A2:**
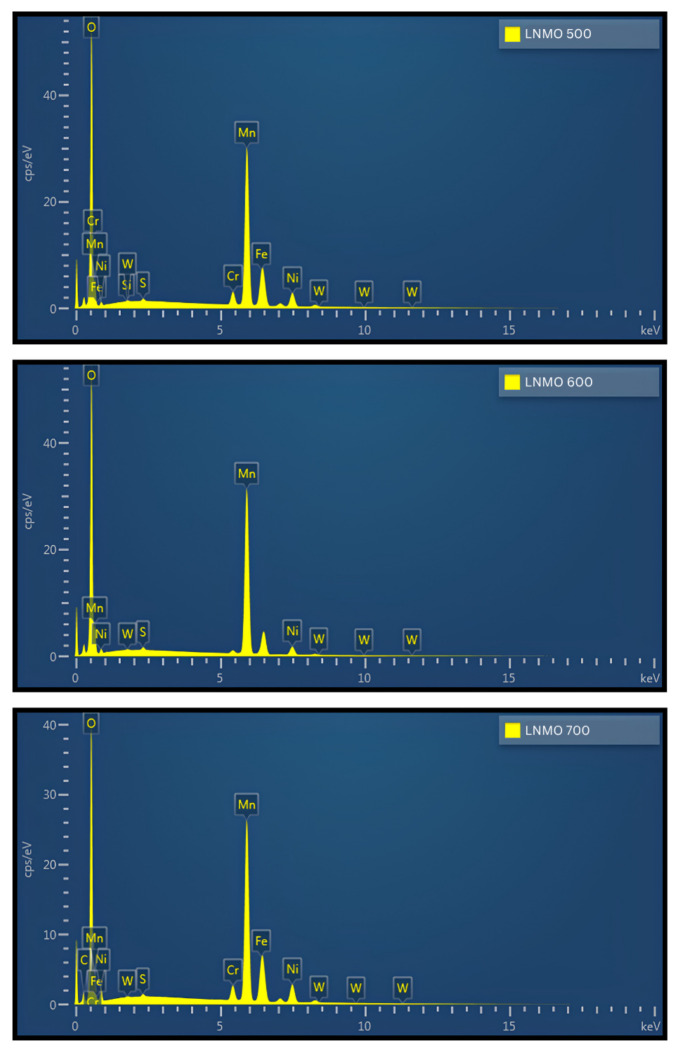
EDS spectra of LNMO samples synthesized at different temperatures.

## Data Availability

The original contributions presented in this study are included in the article. Further inquiries can be directed to the corresponding authors.

## Abbreviations

The following abbreviations are used in this manuscript:

LNMO	Lithium nickel manganese oxide
CEI	Cathode–electrolyte interphase
XRD	X-ray diffraction
SEM	Scanning electron microscopy
EDS	Energy-dispersive spectroscopy
XPS	X-ray photoelectron spectroscopy
CV	Cyclic voltammetry
GDC	Galvanostatic charge–discharge

## Appendix A

### Appendix A.1

**Table A1:** Surface atomic composition of LNMO samples determined by XPS, including all signals under study.

Sample	Atomic Composition (%)					
	Li 1s + Mn 3p	C 1s	O 1s	Mn 2p	Mn 3s	Ni 2p 3/2
LNMO500	58.54	4.52	18.06	8.86	8.16	1.87
LNMO600	60.07	4.52	17.00	8.38	8.16	1.87
LNMO700	56.62	4.66	19.66	8.79	8.43	1.85
